# Serum miRNA Signatures in Cancer Cachexia Depend on Systemic Inflammation

**DOI:** 10.3390/curroncol32110620

**Published:** 2025-11-06

**Authors:** Terese Louise Schmidberger Karlsen, Robin Mjelle, Ola Magne Vagnildhaug, Trude Rakel Balstad, Are Korsnes Kristensen, Jens Erik Slagsvold, Ganna S. Westwik, Hege Elvebakken, Eva Hofsli, Ingunn Hatlevoll, Tora Skeidsvoll Solheim

**Affiliations:** 1 Cancer Clinic, St. Olavs Hospital, 7030 Trondheim, Norway; ola.m.vagnildhaug@ntnu.no (O.M.V.); trude.r.balstad@ntnu.no (T.R.B.); are.kristensen@stolav.no (A.K.K.); eva.hofsli@stolav.no (E.H.); ingunn.hatlevoll@stolav.no (I.H.); tora.s.solheim@ntnu.no (T.S.S.); 2 Department of Clinical and Molecular Medicine, Faculty of Medicine and Health Sciences, NTNU-Norwegian University of Science and Technology, 7491 Trondheim, Norway; robin.mjelle@ntnu.no; 3 Department of Clinical Medicine, Clinical Nutrition Research Group, UiT The Arctic University of Norway, 9019 Tromsø, Norway; 4 Cancer Clinic, Levanger Hospital, Nord-Trøndelag Health Trust, 7601 Levanger, Norway; gannasurzhykova.westwik@helse-nordtrondelag.no; 5 Department of Oncology, Møre & Romsdal Hospital Trust, 6017 Ålesund, Norway; hege.elvebakken@helse-mr.no

**Keywords:** cancer, cachexia, inflammation, biomarkers, MiRNA, colorectal cancer

## Abstract

Cancer cachexia is a complex syndrome marked by involuntary weight and muscle loss, often driven by systemic inflammation. This multicenter, longitudinal observational study investigated circulating microRNA (miRNA) profiles in patients with unresectable locally advanced or metastatic colorectal cancer, comparing those with and without cachexia and inflammation. A total of 168 patients were categorized into four groups based on cachexia and C-reactive protein (CRP) levels. Cachexia was defined using the 2011 consensus criteria, incorporating weight loss, low BMI, and sarcopenia. Patients with both cachexia and systemic inflammation exhibited significantly distinct miRNA profiles as well as poorer overall survival (HR 2.10, *p* < 0.001) compared to patients with neither condition. No significant differences were observed in patients lacking either cachexia or inflammation or both. Inflammatory cachexia emerged as a biologically distinct entity, with 82 differentially expressed miRNAs. The miR-320-family, miR-6087, miR-4488, miR-29a-3p, miR-194-5p, and miR-10a-5p were most altered, several of which are linked to muscle mass, metabolism, lipid, and protein synthesis. These findings highlight the pivotal role of systemic inflammation in cancer cachexia and support its inclusion in diagnostic criteria. Moreover, circulating miRNAs may serve as promising biomarkers for identifying cachexia in cancer patients.

## Simple Summary

Many patients with advanced cancer lose weight and muscle, even if some eat well. This condition, called cancer cachexia, causes a reduced quality of life, increases the side effects of cancer treatment, and shortens life. Cachexia is often linked to systemic inflammation. To better treat this condition, it is essential to understand more about its biological background. We studied small molecules in the blood called microRNAs, which help to control the way genes work. We compared patients depending on whether they had cachexia and/or systemic inflammation. Patients with both cachexia and inflammation had a different microRNA profile and lived for a shorter time. These findings support that inflammation plays an important role in cachexia and should be a part of how we diagnose it. The study also shows that microRNAs in the blood might help doctors detect cachexia earlier and understand how it affects the body.

## 1. Introduction

Cancer cachexia is a wasting syndrome characterized by the loss of skeletal muscle mass and varying degrees of loss of fat mass that cannot be reversed with nutritional support alone. It usually appears in the late stages of cancer, but its prevalence varies extensively with cancer type, and it may sometimes be the first sign of cancer [1]. The syndrome impairs physical function and quality of life and is an independent predictor of mortality [2,3].

Although the knowledge of the mechanisms underlying cancer cachexia is gradually evolving, the pathological processes are still not fully elucidated. Key factors in the pathophysiology are abnormal metabolism and reduced food intake, leading to a negative protein and energy balance. This is driven by a systemic inflammatory response caused by interactions between the host and the tumor [4]. Nevertheless, the definition of cachexia has been discussed for decades. In the international consensus from 2011, criteria for systemic inflammation were not defined, and it was claimed that cachexia could exist without overt systemic inflammation [5]. On the other hand, the Global Leadership Initiative on Malnutrition (GLIM) [6] claimed that cachexia was a chronic, disease-related malnutrition with systemic inflammation.

In the last few decades, microRNAs (miRNAs) have emerged as important molecules for gene regulation. MiRNAs are small (up to 22 nucleotides), non-coding RNA molecules that can bind to messenger RNA (mRNA) and cause either the repression of translation or degradation of the mRNA [7]. Their effect on gene regulation is generally inhibitive and is therefore often referred to as RNA silencing [8]. MiRNAs are found to be regulators of important biological processes such as cellular differentiation and proliferation [9] and they have a key role in the regulation of several inflammatory conditions [10]. Additionally, evidence has also accumulated for dysregulated miRNAs in the development of various diseases, including cancer [11]. MiRNAs can be transported in the circulation within small, membrane-bound particles named exosomes, in other vesicles, or bound to proteins. In this way, the miRNAs can travel to distant tissues and exert their effect in target cells far away from their origin [12].

Studies on cell lines and mice suggest the important role of miRNAs in cachexia [13,14], but studies on miRNAs in humans with cancer cachexia are limited. In addition, most of them include limited patient cohorts, some have examined only one or a few miRNAs, and several of the studies have used muscle or adipose tissue for miRNA analysis [15,16,17,18]. Hence, the results are diverse and challenging to interpret.

As opposed to invasive samples, circulating miRNAs can be assessed from a simple blood sample and therefore have the potential to become easily obtainable biomarkers. To improve the understanding of cachexia pathophysiology, there is a need to validate previous miRNAs associated with cancer cachexia, as well as to find new miRNAs with putative impact on the condition.

Despite increasing focus on the role of inflammation in recent years, the Fearon criteria, or other criteria not addressing the presence of inflammation, are often used in research on cancer cachexia. If, as proposed, systemic inflammation is a necessary component in cancer cachexia, the use of criteria not addressing inflammation status will hinder research, as patients with weight loss, low BMIs, and sarcopenia of other causes than cachexia will be classified as cachectic. A correct classification of cachectic patients is crucial for the characterization and mapping of the molecular pathways underlying cachexia, which is needed both to be able to find biomarkers for the syndrome as well as to be able to identify new therapeutic targets [19].

To our knowledge, there are no studies exploring miRNAs in human cancer cachexia which have used inflammation in addition to standard cachexia criteria to group patients (see Table S1 for prior miRNA studies in cancer cachexia [15,16,17,18,20,21,22,23,24,25,26,27]).

The overall aim of this study is to explore the differential expression of circulating miRNAs in cachectic compared to non-cachectic patients, and to investigate if miRNA expression is influenced by the presence of systemic inflammation.

## 2. Materials and Methods

### 2.1. Study Design and Participants

Between November 2014 and December 2018, 351 patients were included in the metastatic colorectal cancer (mCRC) study from seven cancer clinics in central Norway. This was a prospective observational study including patients with newly diagnosed unresectable locally advanced or metastatic colorectal cancer [28]. Patients that had blood samples available for RNA sequencing and non-missing data for cachexia classification were amenable for analysis.

### 2.2. Data Collection and Assessment

At baseline, demographic information and information on the cancer disease and treatment were registered by health care personnel. The patients underwent medical consultations as clinically indicated, usually bimonthly. Information on status of the cancer disease, cancer treatment, side effects, weight, WHO performance status, and biomarkers such as C-reactive protein (CRP) were registered in a web-based case report form (web-CRF). Blood samples were taken for miRNA analysis at baseline. The Biobank1 information management system (Biobank1, Trondheim, Norway) was used for pseudonymization and secure storage of donor and sample information, including tracking and bar-coding of the samples.

### 2.3. Patient Reported Outcome Measures (PROMs)

The modified Patient-Generated Subjective Global Assessment (mPG-SGA) [29] and the European Organization for Research and Treatment of Cancer (EORTC) Quality of Life questionnaire Core 30 (QLQ-C30) [30] are developed for the self-recording of weight, nutritional status, physical function, and quality of life by cancer patients. Patients in our study completed both these forms.

### 2.4. Muscle Mass Analyses

CT scans performed as part of routine diagnostic workups and treatment evaluation were used for muscle mass analysis. The images of mid-L3 vertebrae were identified by visual inspection, and the skeletal muscle mass from those images was segmented and calculated using the AI-based segmentation tool Data Analysis Facilitation Suite (DAFS) express (Voronoi Health Analytics Incorporated, Vancouver, BC, Canada). The skeletal muscle mass range was set to −29 to 150 Hounsfield units (HUs). Every segmented image was visually inspected and corrected if necessary. A total of 6.7% of the images were manually corrected and a recalculation of body mass was performed using the corrected image in DAFS express.

The muscle area (cm^2^) was adjusted for height squared (m^2^) to give the skeletal muscle index (SMI).

### 2.5. Assessment of Cachexia

The cancer cachexia consensus criteria from 2011 [5] were used to classify patients as cachectic/non-cachectic according to weight loss, low BMI, and/or sarcopenia. Weight loss was assessed over the past six months. Patients with either (1) weight loss > 5%, (2) BMI < 20 and weight loss > 2%, or (3) sarcopenia and weight loss > 2% were defined as being cachectic. Sarcopenia was defined as skeletal muscle index < 55 cm^2^/m^2^ in men and <39 cm^2^/m^2^ in women, in line with the Fearon diagnostic criteria of cancer cachexia [5]. Systemic inflammation was defined as C-reactive protein (CRP) ≥ 10 milligram/liter.

The patients were divided into four different groups as follows:

(1) Non-cachexia without inflammation
(2) Non-cachexia with inflammation
(3) Cachexia without inflammation
(4) Cachexia with inflammation

### 2.6. RNA Extraction, Sequencing, and Calibration

To extract RNA, the miRNeasy Serum/Plasma Kit (ID: 217,184) was used on 200 μL of serum. Small RNA sequencing libraries were developed using 10.5 μL of RNA on the NEXTFLEX^®^ Small RNA-Seq Kit v3 for Illumina^®^ Platforms (PerkinElmer, Waltham, MA, USA). Amplification for 18 PCR cycles as well as sequencing on a HiSeq4000 machine (Illumina, San Diego, CA, USA) [31] were performed on the miRNA libraries. During the initial ligation step of the library preparation protocol, synthetic calibrator RNAs were added. Details concerning RNA isolation, sequencing, and calibration are described previously [31,32].

### 2.7. Differential Expression Analysis

The Limma-voom in R (The R Foundation, Indianapolis, IN, USA) was used for the detection of differentially expressed miRNAs. For inclusion in the analysis, miRNAs had to have at least one count in half of the data. The analyses were adjusted for sex and age. Normalization of miRNAs was performed using normalization factors of the calibrator RNAs [31]. The Benjamini–Hochberg procedure was used to correct for multiple testing and an adjusted significance level of 0.05 was used as the cut-off.

### 2.8. Predicting Targets for Differentially Expressed miRNAs

To explore potential pathways regulated by the differentially expressed miRNAs, the most prominently differing miRNAs were identified. The criteria used for selection were miRNAs with at least two-fold up- or downregulation (log_2_ fold-change ≥ 1), and adjusted *p*-values of less than 0.05. miRWalk was used to identify conserved miRNAs also found in TargetScan, MiRDB, and miRTarBase. Predicted target genes for each of these miRNAs were then retrieved using MiRDB, selecting targets with a target score of above 98.

### 2.9. Gene Ontology Analysis for miRNA Targets

The predicted target genes which had miRDB target scores of above 98 were subjected to a functional enrichment analysis using the g:Profiler (https://biit.cs.ut.ee/gprofiler, accessed on 28 October 2025) (version e113_eg59_p19_f6a03c19). G:Profiler maps genes to Gene Ontology (GO) terms and include pathways from KEGG, Reactome, and WikiPathways [33]. Enrichment was performed using the g:GOSt tool with default statistical settings and multiple testing correction (g:SCS method). The default setting of highlighting driver terms in GO was used. Pathways with adjusted *p*-values < 0.05 were considered significantly enriched. Results were visualized using g:Profiler’s integrated network and bar plot tools to highlight key biological processes and signaling pathways potentially regulated by the miRNAs.

### 2.10. Survival Analysis

Overall survival was calculated from the date of inclusion in the study until death from any cause. The last censoring date was 17 October 2024. Cox proportional hazard analysis was used for estimating hazard ratios (HRs) for death. A univariate analysis with the four groups defined by weight/BMI/sarcopenia and CRP level was first performed. Then the variables age, gender, and liver metastases were added to the model to see if they significantly affected the outcome. Survival was estimated and visualized using Kaplan–Meier analysis.

### 2.11. Analysis and Statistical Considerations

Statistical analysis was performed using STATA version 18.0 (StataCorp LLC, College Station, TX, USA) and R version 4.4.1 (The R Foundation, Indianapolis, IN, USA). An alfa level of 0.05 was used for the assessment of statistical significance.

## 3. Results

### 3.1. Patients

Of the 351 included patients, 193 had blood samples available for RNA sequencing. Of these patients, 25 had missing registrations necessary for cachexia classification, leaving a total of 168 patients amenable to inclusion in the analysis of the association between cachexia, systemic inflammation, and miRNA expression (Figure S1).

Table 1 presents patient baseline characteristics. The mean age of the included patients was 67 years (range: 27–88 years). One of the patients had locally advanced cancer treated with palliative intent, and the rest had metastatic disease. The liver was the organ most often affected by metastases (71% of patients), while 36% of patients had lung metastases.

As shown in Table 1, a total of 94 patients were defined as cachectic. Among those, 74 patients had weight loss > 5%, making it the most frequent cachexia criterion present. Four patients had the combination of BMI < 20 and weight loss > 2%, and all these patients also had weight loss > 5%. Of the patients, 69 were affected by sarcopenia and weight loss > 2%, 49 of which also had weight loss > 5%. The non-cachexia group without systemic inflammation and the non-cachexia group with systemic inflammation consisted of 49 (29%) and 25 (15%) patients, respectively, while the cachexia group without systemic inflammation and the cachexia group with systemic inflammation consisted of 41 (24%) and 53 (32%) patients, respectively. There were no significant differences between the groups concerning age, gender, location of primary tumor, histological grade, or mutation status of the tumor. The group of patients with neither cachexia nor systemic inflammation had a better performance status, while the cachectic patients with systemic inflammation had a poorer performance status (*p* = 0.01). There was also a significant difference in the presence of liver metastases (*p* = 0.02), the cachectic patients with systemic inflammation being the most frequently affected.

### 3.2. miRNA Analyses

**Figure 1:**
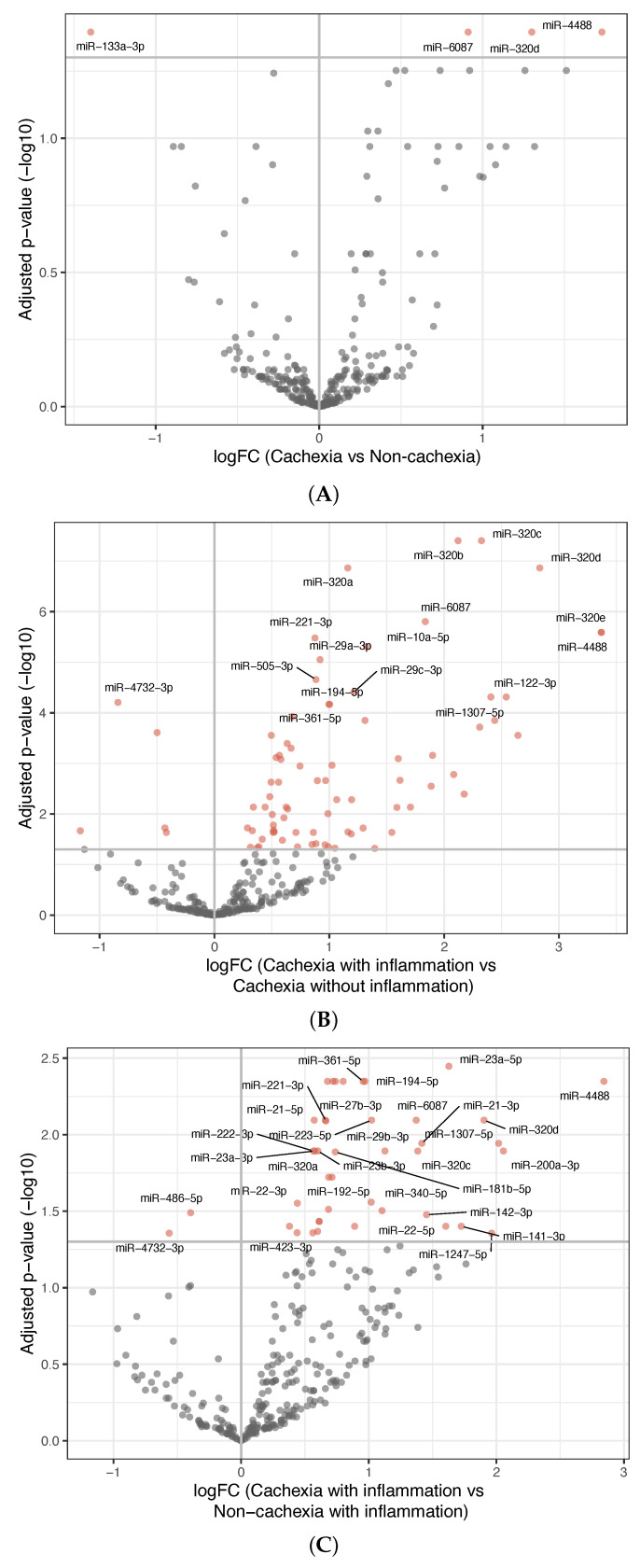
Differentially expressed miRNAs between groups. (**A**) Volcano plot showing differentially expressed miRNAs between patients with cachexia and non-cachexia. The *x*-axis shows the fold-change values (log2) when comparing the two groups. The *y*-axis shows the inverse adjusted *p*-values (−log10). Selected miRNAs are indicated by name. Red indicates significance (Benjamini–Hochberg-adjusted *p*-value < 0.05). (**B**) Similar as in (**A**), comparing inflammatory cachectic patients with non-inflammatory cachectic patients. (**C**) Similar as in (**A**), comparing inflammatory cachectic patients with inflammatory non-cachectic patients. See Supplementary Table S2A–C for a list of the differentially expressed miRNAs and their *p*-values.

Table 2 presents a summary of the analyses performed to explore the differentially expressed miRNAs dependent on phenotype characteristics. Figure 1A depicts the four differentially expressed miRNAs found when comparing all the patients with cachexia with all the non-cachectic patients. MiR-4488, miR-320d, and miR-6087 were upregulated, while miR-133a-3p was downregulated in the patients with cachexia (*p* = 0.04 for all four).

To explore whether miRNA expression was influenced by systemic inflammation, both cachectic and non-cachectic patients were grouped according to elevated or low CRP levels. The comparison between cachectic patients with and without systemic inflammation revealed 80 differentially expressed miRNAs; 75 were upregulated and 5 were downregulated (Figure 1B) in the group of cachectic patients with systemic inflammation. All the three miRNAs upregulated in cachectic versus non-cachectic patients were among these.

To determine if inflammation alone (independent of cachexia) explained the observed miRNA changes, three additional comparisons were made, showing the following:

1. Among patients without cachexia, no significant differences in miRNA expression between those with and without inflammation were found (Figure S2A).
2. Among patients having inflammation, comparing those with and without cachexia revealed 42 differentially expressed miRNAs; 40 were upregulated and 2 were downregulated in the inflammatory cachexia group (Figure 1C). Although fewer than in the comparison between cachectic patients with and without inflammation, most differentially expressed miRNAs overlapped.
3. Among patients without inflammation, no significant differences in miRNA expression were observed between those with and without cachexia (Figure S2B).

**Figure 2:**
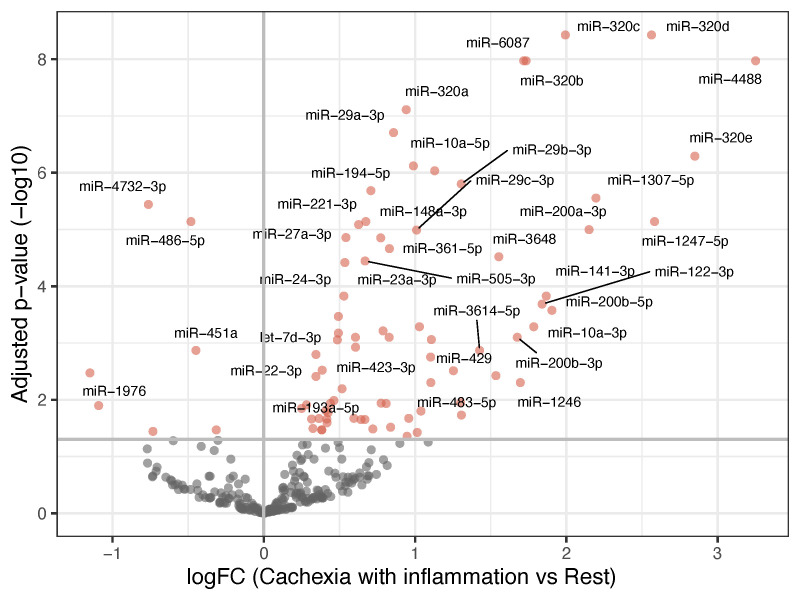
Volcano plot showing differentially expressed miRNAs when comparing patients with cachexia and inflammation with the rest of the patients. The *x*-axis shows the fold-change values (log2) when comparing the two groups. The *y*-axis shows the inverse adjusted *p*-values (−log10). Selected miRNAs are indicated by name. Red indicates significance (Benjamini–Hochberg-adjusted *p*-value < 0.05). See Supplementary Table S3 for a list of the differentially expressed miRNAs and their *p*-values.

These results showed that the cachectic patients with systemic inflammation had a distinct miRNA expression profile compared to all the other groups, while no significant differences were observed between the other three groups. To further explore this distinction, we compared the patients with inflammatory cachexia against all the other patients combined. This analysis identified 82 differentially expressed miRNAs, 75 upregulated and 7 downregulated in the patients with cachexia and systemic inflammation. The most prominent upregulated miRNAs included members of the miR-320-family, as well as miR-6087, miR-4488, miR-29a-3p, miR-194-5p, and miR-10a-5p (Figure 2). Many of these miRNAs overlapped with those identified in the individual pairwise comparisons involving the inflammatory cachexia group.

**Figure 3:**
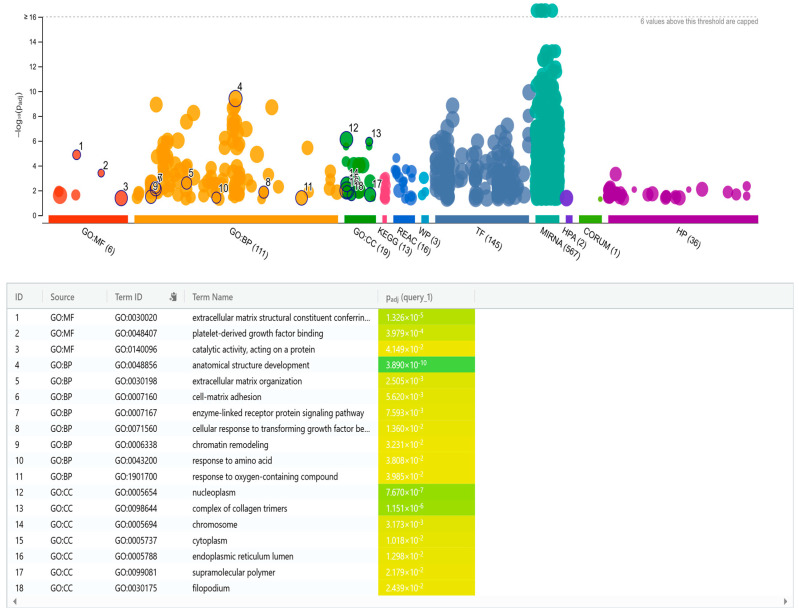
Integrated network and bar plot showing biological processes and signaling pathways potentially regulated by the differentially expressed miRNAs. MF: molecular function, BP: biological process, and CC: cellular component.

Among the differentially expressed miRNAs, 32 showed at least a two-fold change in expression (log_2_ fold-change ≥ 1), either up- or downregulated. Target gene predictions for thirteen of these miRNAs were identified using stringent filtering criteria in miRWalk, and target gene lists were retrieved from miRDB. Focusing on high confidence targets, 145 genes were selected for downstream analysis. Functional enrichment analysis was performed to explore key biological processes and signaling pathways potentially regulated by the miRNAs. Notably, the detected pathways identified were associated with cell growth, differentiation, immune responses, organization of extracellular matrix, as well as proteolytic activity, see Figure 3.

### 3.3. Survival Analyses

During follow-up, 159 patients died. Cox proportional hazard analysis was used to estimate hazard ratios for death. A univariate analysis based on the four patient groups defined by cachexia and inflammation status was performed. The proportional hazards assumption was confirmed using a log-minus-log plot.

**Figure 4:**
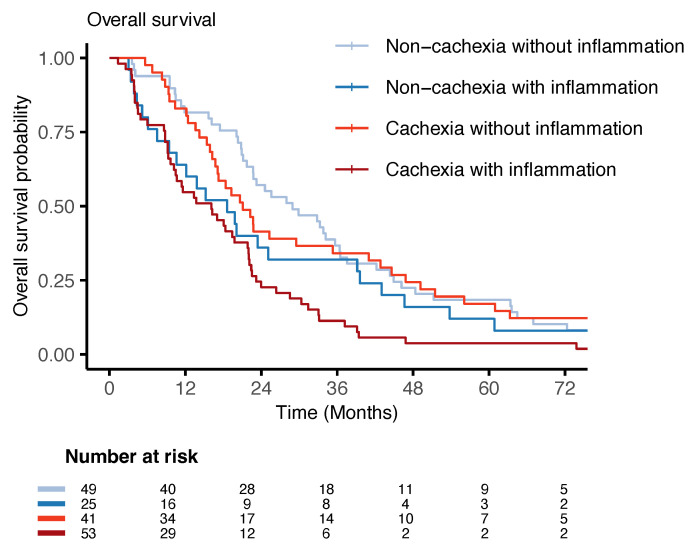
Overall survival plot: Kaplan–Meier survival curves for patients with non-inflammatory non-cachexia, inflammatory non-cachexia, non-inflammatory cachexia, and inflammatory cachexia. The risk table shows the number of individuals at risk at the different time intervals.

In line with the miRNA expression findings, no statistically significant differences in survival were observed between the non-cachexia groups with and without inflammation and the cachexia group without inflammation. However, patients with cachexia and inflammation had a statistically significantly higher hazard for death (HR 2.10, 95% CI 1.40–3.16, and *p* < 0.001) (Table 3) compared to the reference group (non-cachectic patients without inflammation). The survival curves for the different groups are shown in the Kaplan–Meier plot in Figure 4.

To assess if differences in age, gender, and liver metastases between the groups could explain some of the survival differences, these covariates were added in a multivariate model. Adjustment for age, gender, and liver metastases did not significantly alter the hazard ratios (patients with cachexia and inflammation continued to have statistically significantly higher hazards for death (HR 2.08, 95% CI 1.37–3.15, and *p* = 0.001) compared to the reference group (non-cachectic patients without inflammation) (Table S4). The likelihood-ratio test of the univariate versus the multivariate model did not show a better model fit by adding the additional covariates (*p* = 0.09), indicating that the univariate model was sufficient.

## 4. Discussion

This study found that cachectic patients with systemic inflammation exhibited significantly distinct miRNA expression profiles compared to both cachectic patients without inflammation and non-cachectic patients, regardless of inflammatory status. Moreover, those with both cachexia and inflammation had significantly poorer overall survival. In contrast, no significant differences in miRNA profiles or survival were observed among patients without coexisting cachexia and systemic inflammation. Based on our findings, we considered the subgroup with systemic inflammation a distinct cachexia entity. When this group was compared to the rest of the population, 82 differentially expressed miRNAs were detected.

This is the first study to investigate miRNA expression in human cancer cachexia by comparing patients not only by weight loss, muscularity, and BMI, but also taking into account the presence of systemic inflammation. Since cancer cachexia is thought to be driven by an inflammatory reaction to the tumor, it is particularly relevant to explore whether patients with weight loss, low BMI, and/or sarcopenia have miRNA profiles differing not only from patients without these characteristics, but also dependent on whether they have systemic inflammation or not. Such differences would be expected if inflammation plays a central role in the pathophysiology of cachexia.

The results from our study imply an interaction between cachexia and inflammation, as the group with cachexia and systemic inflammation both differed greatly in miRNA expression as well as had an increased hazard for death compared to the other groups. Of special interest is the fact that there were no significant differences in miRNA expression or survival between the cachectic patients without systemic inflammation and the two non-cachexia groups with and without systemic inflammation. This implies that neither systemic inflammation nor the standard cachexia criteria alone can explain the miRNA and survival differences in the inflammatory cachexia group. This also means that, both in terms of miRNA expression and survival, the patients with cachexia without systemic inflammation seem to be more alike with the non-cachexia patients than they are alike with the cachectic patients with systemic inflammation. This could suggest that their weight loss/sarcopenia have other causes than for cachexia patients with systemic inflammation, such as bowel disturbances, nausea, or inactivity. Adding systemic inflammation to the cachexia criteria in line with the GLIM criteria might thus better sort out the patients with “true” cancer cachexia.

At baseline, there were significant differences among the groups in terms of performance status and the presence of liver metastases. The group of patients with neither cachexia nor systemic inflammation demonstrated the best performance status and the lowest occurrence of liver metastases, while the inflammatory cachectic patients exhibited the poorest performance status as well as the highest rate of liver involvement. This is not considered a bias introduced in the grouping process, but rather the reflection of biological differences inherent to the classification based on cachexia and inflammation status. It is well known that cachexia causes functional impairment [5], and a mouse model has shown that the formation of colorectal liver metastases exacerbated cachexia [34]. This is further supported by the observation that including liver metastases, as well as age and gender, as adjustment variables in multivariate survival analysis did not significantly alter the hazard ratios for death when comparing the different cachexia/inflammation groups.

To our knowledge, only one of the 82 differentially expressed miRNAs found in the comparison of cachectic patients with systemic inflammation versus all the other patients is earlier described to be associated with serum changes in patients with cancer cachexia. In a study by Okugawa et al. from 2018 [21], the level of miR-21 in serum was found to be significantly upregulated in colorectal cancer patients with low muscle mass compared to patients with high muscle mass. Furthermore, increased miR-21 levels in serum was shown to be an independent predictive factor for low muscle mass [21]. This finding is in concordance with the results from our study, where miR-21 (both miR-21-5p and miR-21-3p) was found to be significantly upregulated in the cachexia group. The scarcity of other coinciding results can be explained by the fact that there are few human studies on cancer cachexia and serum miRNAs, that most of them examine the expression of one or a few miRNAs only, as well as the fact that none of the other studies have grouped the patients according to inflammation status in addition to standard cachexia criteria.

When comparing our results to studies exploring miRNAs in muscle biopsies, there are more matching results. Narasimhan et al. [15] found eight upregulated miRNAs in pancreatic and colorectal cancer patients with cachexia compared to non-cachectic cancer patients. The dysregulated miRNAs showed prognostic value for overall survival, and predictive value for weight change. In our study, we found five of the same miRNAs to be significantly upregulated in serum, specifically miR-423-5p, let-7d-3p, miR-345-5p, miR-423-3p, and miR-199a-3p. Pathway analysis in the study by Narasimhan et al. [15] indicated that those five upregulated miRNAs were involved in processes directly related to cachexia, namely lipid and protein synthesis and energy homeostasis [15]. Another small study described three upregulated miRNAs and two downregulated miRNAs in the muscles of cachectic patients with non-small-cell lung cancer compared with healthy controls. Pathway analysis identified genes related to muscle atrophy as potential targets of the dysregulated miRNAs [16]. In our study, we found one of these downregulated miRNAs (miR-451-a) to be significantly downregulated in serum.

The connection between differentially expressed miRNAs in serum samples and muscle biopsies is not known, but it is ascertained that miRNAs can travel via the blood stream to reach distant target tissues [12]. The fact that many of the miRNAs found to be dysregulated in muscle biopsies of cachectic patients are also differentially expressed in the serum samples of the cachectic patients in our study could suggest that they play an important role in the pathophysiology of cancer cachexia. If dysregulated miRNAs for the diagnosis of cancer cachexia are found in serum samples, they have the possibility to become applicable biomarkers, in contrast to the much more demanding and invasive muscle biopsies.

As opposed to the studies of dysregulated miRNAs in muscle biopsies, studies exploring dysregulated miRNAs in adipose tissue of cachectic patients do not have results consistent with our findings. Kulyté et al. found four miRNAs to be downregulated and one to be upregulated in the adipose tissue of cachectic gastrointestinal cancer patients compared to weight-stable cancer patients [17]. In our study of serum samples, two of the same miRNAs were dysregulated (miR-483-5p and miR-23a), but they were upregulated instead of downregulated in the cachectic patients. Along the same lines, another study found miR-375 to be significantly downregulated in the adipose tissue of cachectic cancer patients [18], while in our study, miR-375 was upregulated in the patients with cancer cachexia.

It remains unclear why the corresponding dysregulated miRNAs found to be downregulated in adipose tissue in the two aforementioned studies were upregulated in serum in our study. One possible explanation is that these miRNAs, although downregulated in adipose tissue, might be released into circulation during adipolytic processes, leading to elevated serum levels. Further studies are needed to determine whether this is the case and to clarify the potential role of these miRNAs in cancer cachexia.

When exploring potential pathways regulated by the most differentially expressed miRNAs, pathways involved in cell growth, cell differentiation, immune responses, organization of extracellular matrix, as well as proteolytic activity were identified. These processes are known to be highly relevant in cachexia [4,35,36,37] and could therefore support the clinical and biological relevance of our findings. Nevertheless, they are also involved in various other conditions, and are therefore not specific to cachexia. Consequently, large studies on human cancer patients are needed to validate these results and identify the most suitable miRNA biomarkers for cancer cachexia. In this study, the members of the miR-320-family, as well as miR-6087, miR-4488, miR-29a-3p, miR-194-5p, and miR-10a-5p were the most prominently upregulated miRNAs and might therefore have the best potentials as cancer cachexia biomarkers.

### Limitations

The present study is, to our knowledge, one of the largest studies investigating serum miRNA expression in cancer cachexia, considering both the number of miRNAs explored as well as of patients included. It is also the first study of miRNAs in human cancer cachexia to group patients based on both standard cachexia criteria as well as on inflammation status. However, there is a risk of selection bias, as the patients without registered CT scans or weight registrations were excluded. These patients may potentially have reduced physical performance and poor prognosis, although no significant differences in age, performance status, or overall survival were found when comparing the included with the excluded patients (data not reported). It is also worth noting that CRP, used as marker for systemic inflammation, is not only affected by inflammation, but also by transient infections. Some patients with elevated CRP levels might thus have had a subclinical infection and not chronic disease-related systemic inflammation, and might in that sense be misclassified. Conversely, using a fixed CRP cut-off of 10 mg/l might lead to misclassification in the other direction, as some patients with chronic low-grade inflammation may not exhibit CRP levels above this threshold. To improve accuracy in future studies, incorporating cytokine panels or additional inflammatory markers alongside CRP could provide a more comprehensive assessment of inflammation status.

## 5. Conclusions

This study demonstrates that cachectic patients with systemic inflammation exhibit a distinct miRNA expression profile when compared to all the other groups, including those with cachexia but without inflammation. Patients in this subgroup also experienced the poorest overall survival outcomes. In contrast, there were no significant differences in miRNA expression or survival between the other groups. This indicates that systemic inflammation is a critical part of the pathophysiology of cancer cachexia and supports the inclusion of CRP-based inflammation assessments in the definition of cancer cachexia. Pathway analysis of the most differentially expressed miRNAs in patients with cachexia and inflammation identified potential gene targets involved in cell growth and differentiation, immune responses, and proteolytic activity. These processes are known to be important in cachexia, though they are not exclusive to the condition. Further research is therefore needed to clarify the specific mechanistic contributions of each differentially expressed miRNA in the development and progression of cachexia, and to determine how they can contribute to future therapeutic strategies or serve as diagnostic biomarkers.

## Figures and Tables

**Table 1:** Patient baseline characteristics.

	Non-Cachexia Without Inflammation *n* = 49 (29%)	Non-Cachexia with Inflammation *n* = 25 (15%)	Cachexia Without Inflammation *n* = 41 (24%)	Cachexia with Inflammation *n* = 53 (32%)	Total *n* = 168 (100%)	*p*-Value
**Sex**						0.76 ^a^
Male	36 (73%)	17 (68%)	26 (63%)	35 (66%)	114 (68%)	
Female	13 (27%)	8 (32%)	15 (37%)	18 (34%)	54 (32%)	
**Age, mean (SD)**	66 (11)	69 (6.6)	67 (12)	67 (10)	67 (10)	0.53 ^b^
**Cachexia criterion**						
Weight loss > 5% last six months	-	-	33 (80%)	41 (77%)	74 (44%)	
BMI < 20 and weight loss > 2%	-	-	1 (2.4%)	3 (5.7%)	4 (2.4%)	
Sarcopenia and weight loss > 2%	-	-	27 (87%)	42 (91%)	69 (46%)	
**WHO performance status**						0.01 ^c^
0	30 (61%)	13 (52%)	18 (45%)	16 (30%)	77 (46%)	
1	17 (35%)	7 (28%)	16 (40%)	28 (53%)	68 (41%)	
≥2	2 (4.1%)	5 (20%)	6 (15%)	9 (17%)	22 (13%)	
**Location of primary tumor**						0.54 ^a^
Right-sided tumor	15 (31%)	9 (36%)	15 (38%)	22 (45%)	61 (37%)	
Left-sided tumor	34 (69%)	16 (64%)	25 (63%)	27 (55%)	102 (63%)	
**Metastases**						
Liver	30 (61%)	18 (72%)	26 (63%)	46 (87%)	120 (71%)	0.02 ^a^
Lung	19 (39%)	14 (56%)	10 (24%)	17 (32%)	60 (36%)	0.06 ^a^
Peritoneal	10 (20%)	3 (12%)	13 (32%)	13 (25%)	39 (23%)	0.30 ^a^
Non-regional lymph nodes	11 (22%)	3 (12%)	14 (34%)	15 (28%)	43 (26%)	0.22 ^a^
**Histological grade**						0.15 ^a^
Highly or moderately diff	31 (86%)	13 (65%)	24 (80%)	21 (66%)	89 (75%)	
Poorly or undiff	5 (14%)	7 (35%)	6 (20%)	11 (34%)	29 (25%)	
**RAS status**						0.46 ^a^
Mutation KRAS or NRAS	17 (37%)	14 (56%)	18 (45%)	24 (48%)	73 (45%)	
WT KRAS and NRAS	29 (63%)	11 (44%)	22 (55%)	26 (52%)	88 (55%)	
**BRAF status**						0.56 ^a^
Mutation BRAF	6 (13%)	4 (16%)	10 (25%)	10 (20%)	30 (19%)	
WT BRAF	39 (87%)	21 (84%)	30 (75%)	40 (80%)	130 (81%)	

^a^ Chi-square test; ^b^ one-way ANOVA test; and ^c^ Kruskal–Wallis test. Abbreviations: Diff, differentiated; RAS status, rat sarcoma status; KRAS, Kirsten rat sarcoma virus; NRAS, neuroblastoma RAS viral oncogene homolog; WT, wild-type; and BRAF: B-RAF proto oncogene.

**Table 2:** Differentially expressed miRNAs when comparing groups defined by cachexia and inflammation status.

Groups Compared	Differentially Expressed miRNAs
Cachexia vs. non-cachexia	4 in total; 3 upregulated, 1 downregulated in the cachexia group (Table S2A)
Cachexia with inflammation vs. cachexia without inflammation	80 in total; 75 upregulated, 5 downregulated in the inflammatory cachexia group (Table S2B)
Non-cachexia with inflammation vs. non-cachexia without inflammation	None
Cachexia with inflammation vs. non-cachexia with inflammation	42 in total; 40 upregulated, 2 downregulated in the inflammatory cachexia group (Table S2C)
Cachexia without inflammation vs. non-cachexia without inflammation	None
Cachexia with inflammation vs. non-cachexia without inflammation	46 in total; 37 upregulated, 9 downregulated in the inflammatory cachexia group (Table S2D)
Cachexia without inflammation vs. non-cachexia with inflammation	None
Cachexia with inflammation vs. all other patients	82 in total; 75 upregulated, 7 downregulated in the inflammatory cachexia group (Table S3)

**Table 3:** Overall survival for groups defined by cachexia and inflammation status.

	Hazard Ratio	*p*-Value	95% Confidence Interval
Reference group: Non-cachexia without inflammation			
Non-cachexia with inflammation	1.37	0.22	0.83–2.27
Cachexia without inflammation	1.12	0.60	0.73–1.73
Cachexia with inflammation	2.10	<0.001	1.40–3.16

## Data Availability

The original contributions presented in this study are included in the article/Supplementary Materials. Further inquiries can be directed to the corresponding author.

## Supplementary Materials

The following supporting information can be downloaded at https://www.mdpi.com/article/10.3390/curroncol32110620/s1, Figure S1: Consort diagram; Figure S2: (A) Differentially expressed miRNAs comparing inflammatory non-cachectic patients with non-inflammatory non-cachectic patients; (B) Differentially expressed miRNAs comparing non-inflammatory cachectic patients with non-inflammatory non-cachectic patients; Table S1: Prior miRNA studies in human cancer cachexia [15,16,17,18,20,21,22,23,24,25,26,27]; Table S2: (A) Differentially expressed miRNAs when comparing patients with versus without cachexia; (B) Differentially expressed miRNAs when comparing cachectic patients with inflammation versus cachectic patients without inflammation; (C) Differentially expressed miRNAs when comparing cachectic patients with inflammation versus non-cachectic patients with inflammation; (D) Differentially expressed miRNAs when comparing cachectic patients with inflammation versus non-cachectic patients without inflammation; Table S3: Differentially expressed miRNAs when comparing cachectic patients with inflammation versus all other patients; Table S4: Multivariate survival analysis.
