# Supplementary material for: Serum miRNA Signatures in Cancer Cachexia Depend on Systemic Inflammation
Source: Curr Oncol. 2025 Nov 6;32(11):620. doi: 10.3390/curroncol32110620 (PMC12651830; doi:10.3390/curroncol32110620)
Supplement: Supplementary file 1 [file curroncol-32-00620-s001.zip › supplementary figures.pdf]

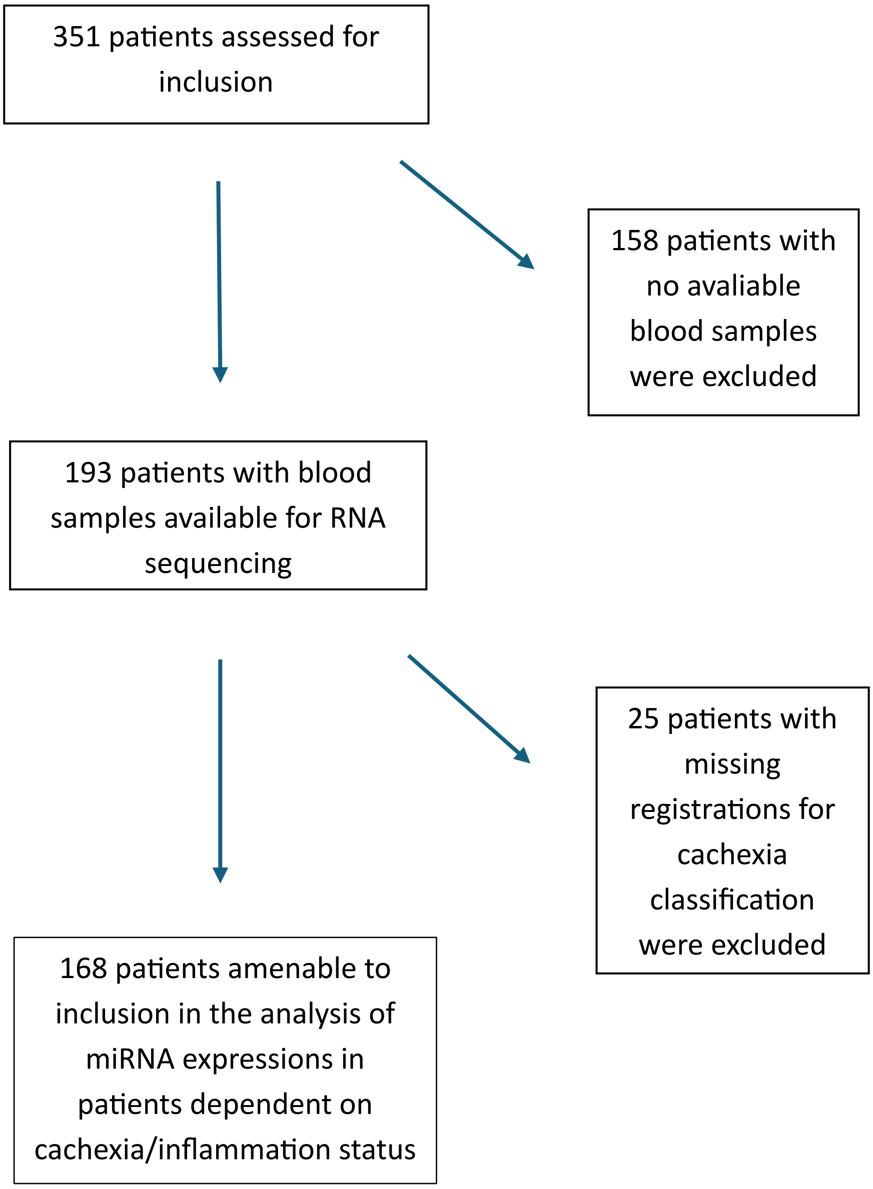

Figure S1. Consort diagram.

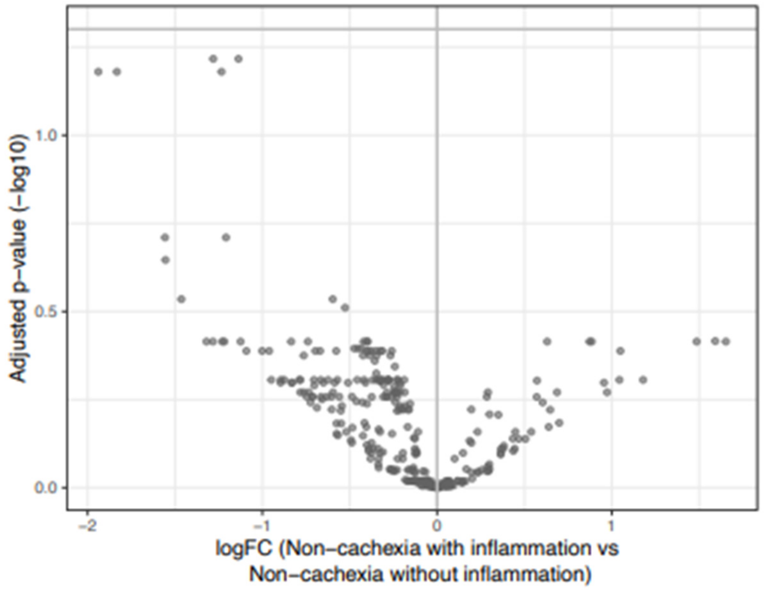

(A)

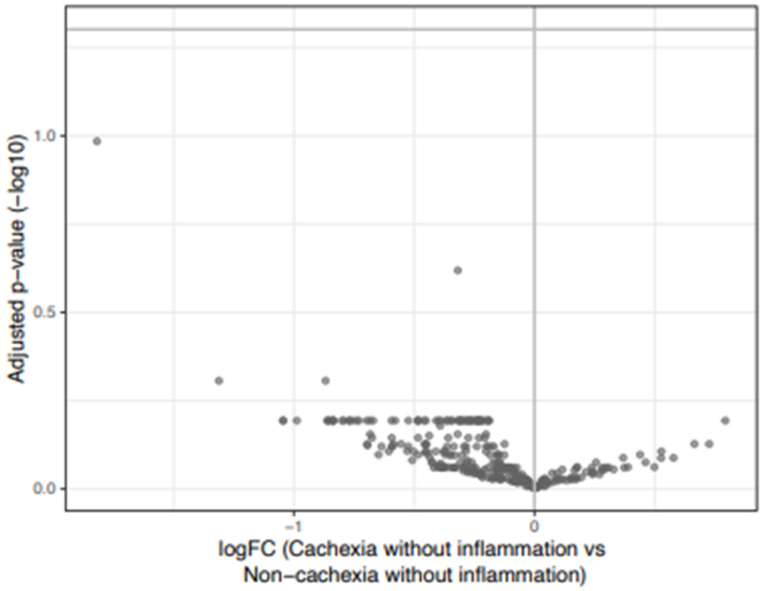

(B)

Figure S2. (A) Differentially expressed miRNAs comparing inflammatory non-cachectic patients with non-inflammatory non-cachectic patients; (B) Differentially expressed miRNAs comparing non-inflammatory cachectic patients with non-inflammatory non-cachectic patients.
