# Supplementary material for: Serum miRNA Signatures in Cancer Cachexia Depend on Systemic Inflammation
Source: Curr Oncol. 2025 Nov 6;32(11):620. doi: 10.3390/curroncol32110620 (PMC12651830; doi:10.3390/curroncol32110620)
Supplement: Supplementary file 1 [file curroncol-32-00620-s001.zip › Table S1.pdf]

Table S1. Prior miRNA studies in cancer cachexia.

Study	Patients	Cancer cachexia criteria	Studied miRNA	Results	Dysregulated miRNAs
Kulyté, 2014 (1)	N= 21; 10 with cancer cachexia and 11 weight-stable gastrointestinal cancer patients	1) Weight loss of ≥5% during the previous three months or<br>2) ≥ 10 % during the previous six months	Total miRNA from abdominal subcutaneous adipose tissue	Five differentially expressed miRNAs (four downregulated, one upregulated).<br>MiR-483-5p, miR-23a, miR-744, miR-99b were significantly downregulated in cachexia patients, but did not correlate with lipolysis ex vivo.<br>MiR-378 was significantly upregulated and strongly and positively correlated with catecholamine-stimulated lipolysis in adipocytes	Downregulated: miR-483-5p, miR-23a, miR-744, miR-99b<br><br>Upregulated: miR-378
Narasimhan, 2017 (2)	N=42 patients with pancreatic and colorectal cancer with liver metastases;<br>22 patients with and 20 without cachexia	1) ≥5% weight loss last six months or<br>2) BMI <20 with any degree of weight loss >2 % or<br>3) lumbar skeletal sarcopenia	Total miRNA from m.rectus abdominis biopsies	Eight miRNAs upregulated in cachectic patients.<br>Pathway analysis indicated that the upregulated miRNAs were involved in adipogenesis, myogenesis, inflammation, innate immune response	Upregulated miRs: hsa-miR-3184-3p, hsa-miR-423-5p, hsa-let-7d-3p, hsa-miR-1296-5p, hsa-miR-345-5p, hsa-miR-532-5p, hsa-miR-423-3p and hsa-miR-199a-3p
Powrózek, 2018 (3)	N = 70 patients with head and neck cancer	Weight loss ≥ 5 %	miR-130a in plasma	Patients with low miRNA-130a levels had significantly higher risk of being moderately or severely malnourished (SGA-B+C) compared	Downregulated: miR-130a

				to cases with high miRNA-130a expression	
Okugawa, 2018 (4)	N = 167 patients with colorectal cancer	Psoas muscle mass index (PMI)= cross-sectional area of bilateral psoas muscle/ height^2. Low PMI was regarded as a proxy for low muscle volume	miR-21 in cancer tissues (n=134) and serum samples (n=153)	Serum miR-21-expression, but not tissue miR-21 expression, was significantly increased in patients with low PMI compared to patients with high PMI. Multivariate logistic regression analysis also showed increased miR-21 to be an independent risk factor for low PMI	miR-21
Okugawa, 2019 (5)	N = 183 patients with colorectal cancer (CRC)	Psoas muscle mass index (PMI) = cross-sectional area of bilateral psoas muscle/ height^2. Low PMI was regarded as a proxy for low muscle volume	miR-203 in cancer tissues and serum samples	Elevated levels of miR-203 associated with low psoas muscle index (PMI) and was found to be an independent risk factor for myopenia	miR-203
Van De Worp, 2020 (6)	N= 16; 8 patients with NSCLC and cachexia and N= 8 healthy controls.<br>The six top-ranked miRNAs were confirmed using reverse transcription-qRT-PCR in an extended group (n = 48) of treatment-naïve NSCLC with (n = 15) and without cachexia (n = 11) and healthy controls (n = 22)	Consensus criteria from 2011, not further specified	Total miRNA from m.vastus lateralis biopsies	28 differentially expressed miRNAs in cachexia compared to healthy controls (5 upregulated, 23 downregulated).<br>Of the six top-ranked miRNAs; three were upregulated, and two downregulated in the muscle of cachectic patients compared to healthy controls. The differentially expressed miRNAs were not found to have prognostic value for overall survival	Upregulated miRs: miR-424-5p, miR-424-3p and miR-450a<br><br>Downregulated miRs: miR-451a and miR-144-5p

Yehia, 2021 (7)	N = 203 patients with local/advanced or metastatic pancreatic and NSCLC, of which 94 were non-cachectic and 109 were cachectic	Weight loss of >5%, BMI<20 and weight loss of >2% or sarcopenia and weight loss >2%	miR-155 in serum samples	miR-155 was highly statistically significantly different in cachectic vs non-cachectic patients. Overexpression of miR-155 was also increasing with increasing phases of cachexia (pre-cachexia, cachexia and refractory cachexia)	miR-155
Sun, 2021 (8)	N = 6 patients with gastric cancer, three with and three without cancer cachexia.<br>For further analyses: 60 additional gastric cancer patients (30 CAC and 30 non-CAC)	Weight loss of >5% last six months, BMI<20 and weight loss of >2% last six months	Total miRNA from subcutaneous adipose tissue in 6 patients. 11 differentially expressed miRNAs with low p-value (CAC vs non-CAC) analyzed in 60 additional patients	miR-410-3p was the miRNA most differentially expressed in CAC- vs non-CAC-patients. Further analysis found a high correlation between levels of miR-410-3p expression and weight loss	miR-410-3p
Krauss, 2023 (9)	N=35 patients with GI cancer; 12 weight stable (non-CCx) and 23 cachectic (CCx)<br><br>For the validation analyses, samples from patients from the three different studies served as control patients without cancer (unknown number):	Weight loss ≥ 5 % last 6 months	158? (from abstract)/169? (from methods-section) miRNAs were profiled in four pooled serum samples; pools of 2 men ±CCx and pools of 2 women ±CCx<br><br>Five miRNAs (miR-19b-3p, miR-122-5p, miR-142-5p, miR-194-5p, miR-486-5p) showed significant differences between the two conditions. For validation, those five	Serum: Expression levels of miR-122-5p different in CCx and non-CCx patients. Correlations of miR-122-5p expression levels and the percentage of weight loss 6 months before surgery showed significant negative relationship.<br><br>Muscle: 6 downregulated miRNAs in CCx compared with non-CCx patients: miR-19b-3p, miR-27b-3p, miR-103a-3p, miR-142-5p, miR-199a-3p and miR-424-5p. When correlated with weight loss the last six months, only miR-27b-3p showed a significantly negative relationship. MiR-27b-3p was not	Serum: miR-122-5p<br><br>Liver: no differences in expression levels<br><br>Muscle: miR-19b-3p, miR-27b-3p, miR-103a-3p, miR-142-5p, miR-199a-3p and miR-424-5p lower expressed in CCx compared with non-CCx.<br>(after validation: miR-27b-3p. But miR-27b-3p not expressed at lower levels in patients with CCx compared with control group!)

			miRNAs as well as five miRNAs previously reported to be related to CCx (miR-27b-3p, miR-103a-3p, miR-199a-3p, miR-375 and miR-424-5p) were analyzed in serum, visceral and subcutaneous adipose tissue, liver and muscle tissue (of patients with and without CCx and a healthy control group)	significantly different in CCx patients compared with the healthy control group.<br><br>Visceral adipose tissue (VAT): The expression of eight of the ten miRNAs (exception for miR-122-5p and miR-486-5p) was significantly lower in VAT of CCx compared to non-CCx.<br>miR-375 and miR-424-5p were correlated with percentage weight loss the last 6 months.<br>Only miR-375 also had significantly lower expression compared to the healthy control group.<br><br>Liver tissue: no differentially expressed miRNAs were found	Visceral adipose tissue: miR-375
Molfino, 2023 (10)	N= 40;<br>25 patients with gastrointestinal cancer (9 colorectal, 7 gastric, 9 pancreatic cancer) and 15 healthy controls	Patients divided in two groups according to sex-specific tertiles of SMI (those at the lowest tertile of SMI vs those with the middle and highest tertiles of SMI	In serum samples:<br>7 selected miRNAs based on previous reports:<br>miR-15b-5p, miR-21-5p, miR-29a-3p, miR-29b-3p, miR-133a-3p, miR-206, miR-486-5p<br><br>In m.rectus abdominis biopsies: RNA sequencing	In serum samples: miR-133a-3p levels were increased in the moderate-high muscularity group compared to the low muscularity group. When stratified according to sex, no differences in the levels of the examined miRNAs in the two muscularity groups seen for women.<br><br>(Muscle biopsies: 10 upregulated, 56 downregulated miRNAs in cancer patients vs controls, not commented on muscularity)	miR-133a-3p upregulated in serum of patients in the moderate-high muscularity group

Tambaro, 2024 (11)	N=51;<br>n = 37 patients with gastrointestinal cancer (colorectal (n=32), gastric (n=4) and pancreatic (n=1)) and n=14 healthy controls	Consensus criteria from 2011, not further specified	Serum: 5 selected miRNAs:<br>miR-26a, miR-128, miR-144, miR-155 and miR-181a	None of the five miRNAs examined in this study were differentially expressed in cachectic vs non-cachectic patients	None
Hu, 2025 (12)	N = 34;<br>23 gastrointestinal cancer patients and 11 healthy controls	(1) Presence of underlying disease<br>(2) Weight loss >2%/3–6 months or low BMI (<21 kg/m2)<br>One or more of the following:<br>(1) Anorexia<br>(2) Decreased grip strength<br>(3) Elevated CRP	Skeletal muscle and tumor tissue: Total miRNA	Three miRNAs (miR-203a-3p, miR-203b-3p, miR-203b-5p) were upregulated in both tumor and muscle tissues of cachectic PC patients compared to non-cachectic patients	Three upregulated miRNAs: miR-203a-3p, miR-203b-3p, miR-203b-5p

References:

1. Kulyté A, Lorente-Cebrián S, Gao H, Mejhert N, Agustsson T, Arner P, et al. MicroRNA profiling links miR-378 to enhanced adipocyte lipolysis in human cancer cachexia. Am J Physiol Endocrinol Metab. 2014;306(3):E267–74.
2. Narasimhan A, Ghosh S, Stretch C, Greiner R, Bathe OF, Baracos V, et al. Small RNAome profiling from human skeletal muscle: novel miRNAs and their targets associated with cancer cachexia. J Cachexia Sarcopenia Muscle. 2017;8(3):405–16.
3. Powrózek T, Mlak R, Brzozowska A, Mazurek M, Gołębiowski P, Małecka-Massalska T. miRNA-130a Significantly Improves Accuracy of SGA Nutritional Assessment Tool in Prediction of Malnutrition and Cachexia in Radiotherapy-Treated Head and Neck Cancer Patients. Cancers (Basel). 2018;10(9).
4. Okugawa Y, Yao L, Toiyama Y, Yamamoto A, Shigemori T, Yin C, et al. Prognostic impact of sarcopenia and its correlation with circulating miR-21 in colorectal cancer patients. Oncol Rep. 2018;39(4):1555–64.
5. Okugawa Y, Toiyama Y, Hur K, Yamamoto A, Yin C, Ide S, et al. Circulating miR-203 derived from metastatic tissues promotes myopenia in colorectal cancer patients. J Cachexia Sarcopenia Muscle. 2019;10(3):536–48.
6. van de Worp W, Schols A, Dingemans AC, Op den Kamp CMH, Degens J, Kelders M, et al. Identification of microRNAs in skeletal muscle associated with lung cancer cachexia. J Cachexia Sarcopenia Muscle. 2020;11(2):452–63.
7. Yehia R, Schaalan M, Abdallah DM, Saad AS, Sarhan N, Saleh S. Impact of TNF-α Gene Polymorphisms on Pancreatic and Non-Small Cell Lung Cancer-Induced Cachexia in Adult Egyptian Patients: A Focus on Pathogenic Trajectories. Front Oncol. 2021;11:783231.
8. Sun D, Ding Z, Shen L, Yang F, Han J, Wu G. miR-410-3P inhibits adipocyte differentiation by targeting IRS-1 in cancer-associated cachexia patients. Lipids Health Dis. 2021;20(1):115.
9. Krauss T, Heisz S, Honecker J, Prokopchuk O, Martignoni M, Janssen KP, et al. Specific miRNAs are associated with human cancer cachexia in an organ-specific manner. J Cachexia Sarcopenia Muscle. 2023;14(3):1381–94.
10. Molfino A, Beltrà M, Amabile MI, Belli R, Birolo G, Belloni E, et al. Small non-coding RNA profiling in patients with gastrointestinal cancer. J Cachexia Sarcopenia Muscle. 2023;14(6):2692–702.
11. Tambaro F, Imbimbo G, Pace V, Amabile MI, Rizzo V, Orlando S, et al. Circulating adipose-tissue miRNAs in gastrointestinal cancer patients and their association with the level and type of adiposity at body composition analysis. Front Mol Biosci. 2024;11:1449197.
12. Hu Y, Hu Y, Zhang S, Guo Y, Wang F, Du Y, et al. Tumor-derived miR-203a-3p potentiates muscle wasting by inducing muscle ferroptosis in pancreatic cancer. Cancer Lett. 2025;614:217523.
