# Supplementary material for: Serum miRNA Signatures in Cancer Cachexia Depend on Systemic Inflammation
Source: Curr Oncol. 2025 Nov 6;32(11):620. doi: 10.3390/curroncol32110620 (PMC12651830; doi:10.3390/curroncol32110620)
Supplement: Supplementary file 1 [file curroncol-32-00620-s001.zip › Table S4.pdf]

Table S4. Multivariate survival analysis.

	Hazard ratio	p-value	95% confidence interval
Reference group: Non-cachexia without inflammation			
Non-cachexia with inflammation	1.30	0.30	0.79 - 2.16
Cachexia without inflammation	1.08	0.73	0.70 - 1.66
Cachexia with inflammation	2.08	0.001	1.37 - 3.15
Age	1.02	0.02	1.00 - 1.03
Female sex	1.30	0.13	0.92 - 1.83
Livermetastases	0.92	0.64	0.64 – 1.31
